# Physicochemical Characterization, Antioxidant, and Proliferative Activity of Colombian Propolis Extracts: A Comparative Study

**DOI:** 10.3390/molecules29071643

**Published:** 2024-04-06

**Authors:** Diana Marcela Buitrago, Sandra J. Perdomo, Francisco Arturo Silva, Willy Cely-Veloza, Gloria Inés Lafaurie

**Affiliations:** 1 Unidad de Investigación Básica Oral—UIBO, Facultad de Odontología, Universidad El Bosque, Bogotá 110121, Colombia; 2 Cellular and Molecular Immunology Group-INMUBO, Facultad de Odontología, Universidad El Bosque, Bogotá 110121, Colombia; perdomosandraj@unbosque.edu.co; 3 Apisred SAS, Otás 414028, Colombia; apisredsas@gmail.com; 4 Área Bioclínica, Facultad de Odontología, Universidad El Bosque, Bogotá 110121, Colombia; wcelyv@unbosque.edu.co; 5 Bioorganic Chemistry Laboratory, Facultad de Ciencias Básicas y Aplicadas, Universidad Militar Nueva Granada, Cajica 250247, Colombia

**Keywords:** propolis, cell proliferation, chemical composition, physicochemical characterization, fibroblasts

## Abstract

Propolis extracts have been widely studied due to their popularity in traditional medicine, presenting incredible biodiversity. This study aimed to analyze propolis extracts’ phytochemical, physicochemical, and biological activities from four different biogeographic zones of the Huila region (Colombia). The raw material samples were collected by the scraping method and the ethanolic extracts (EEPs) were obtained by cold maceration with ethanol (96%). The physicochemical and sensory characterization was carried out according to the protocols recommended by the Brazilian Ministry of Agriculture and the main components of the EEPs were identified by LC-HRMS analysis. The determination of total phenols and flavonoids was carried out using colorimetric techniques. The antioxidant activity, cytotoxicity, and cell cycle regulation analyses in L929 and HGnF cells were evaluated using DPPH, Alamar Blue, and 7-amino actinomycin D (7-AAD) assays. The propolis samples presented an average yield of 33.1%, humidity between 1.6 and 2.8%, melting point between 54 and 62 °C, ashes between 1.40 and 2.19%, and waxes of 6.6–17.9%, respectively. The sensory characteristics of all samples were heterogeneous, complying with the quality specifications established by international standards. The polyphenolic and total flavonoid content was representative in the samples from Quebradon (255.9 ± 9.2 mg GAE/g, 543.1 ± 8.4 mg QE/g) and Arcadia (543.1 ± 8.4 mg GAE/g, 32.5 ± 1.18 g QE/g) (*p* < 0.05) that correlated with high antioxidant activity (Quebradon: 37.2 ± 1.2 µmol/g, Arcadia: 38.19 ± 0.7 µmol/g). In the chemical composition analysis, 19 compounds were characterized as phenolic acids and flavonoids, the most representative being chrysoeriol-*O*-methyl-ether, ellagic acid, and 3,4-*O*-dimethylcaffeic acid. Regarding biological activity, Quebradon and Arcadia propolis presented low toxicity with IC_50_ of 2.83 ± 2.3 mg/mL and 4.28 ± 1.4 mg/mL in HGnF cells, respectively, and an arrest of the cell cycle in the G2/M phase of 71.6% and 50.8% compared to the control (11.9%) (*p* < 0.05). In general, the results of this study contribute to the identification of valid quality criteria to evaluate Colombian propolis, contributing to its study and chemical and biological characterization as a source of raw material for industrial and pharmaceutical use. In addition, Quebradon and Arcadia propolis can be important sources of bioactive molecules for the development of new drugs.

## 1. Introduction

Propolis contains natural ingredients and is produced by bees using substances collected from poplar and conifer trees, clusters of flowers or trees, and hive cells [1]. Propolis extracts have a complex chemical composition that depends on geographical location due to variations in exudates or secretions produced by the plants that grow around the hives and >300 secondary metabolites, including flavonoids, phenylpropanoids, terpenes, stilbenes, lignans, coumarins, and their prenylated derivatives [2,3,4]. However, the most significant metabolite of propolis is flavonoids, which are responsible for their antimicrobial, antioxidant, and anti-inflammatory actions [5,6,7].

In traditional medicine, propolis is used to prevent and treat colds and heal skin wounds [8,9], ulcers [10], rheumatoid arthritis [11], brain and neurological disorders and injuries [12], cardiovascular disease [13], and diabetes [14]. It also highlights the different biological activities against which raw propolis extracts and fractions of different polarities have been evaluated as antioxidant [6,15], anti-inflammatory [6,15], antiallergic [15], and antibacterial agents [5,16] and have been widely used in bone restoration and processes related to dental mineralization and oral healthcare [17].

Propolis has other physicochemical and organoleptic characteristics that depend mainly on the hive’s geographical area, climatic conditions, and botanical origin [18]. One of the most widely studied propolis originates from Brazil. However, these differ from European propolis in that only three phenolic acids (gallic, caffeic, and coumaric acid), a derivative of cinnamic acid (artepilin C), and a flavonoid (pinocembrin) have been identified [19,20].

Several chemical types of propolis have been described based on their plant source. Reliable criteria for the chemical standardization of different propolis types are needed, but such generally accepted criteria still need to be created. The chemical profile of “poplar” propolis has been characterized by the following parameters: total flavone and flavanol content, total flavanone and dihydro-flavanol content, and total phenolics content, which correlate with the biological activity. However, it is necessary to achieve standardization of other propolis types [21]. Colombia is the second most biodiverse country globally and has different thermal floors [22]. Although the use of propolis in traditional medicine is more widespread in Colombia than in other countries, studies on its chemical composition, physicochemical standardization, and biological activity that would guarantee its quality, safety, and efficacy still need to be completed. Therefore, this study evaluated the chemical composition, physicochemical parameters, antioxidant activity, viability, and cellular proliferation of four propolis obtained in different geographical areas of southwest Colombia, in the Huila Region.

## 2. Results and Discussion

### 2.1. Sensorial Analysis of Propolis Extracts

**Figure 1:**
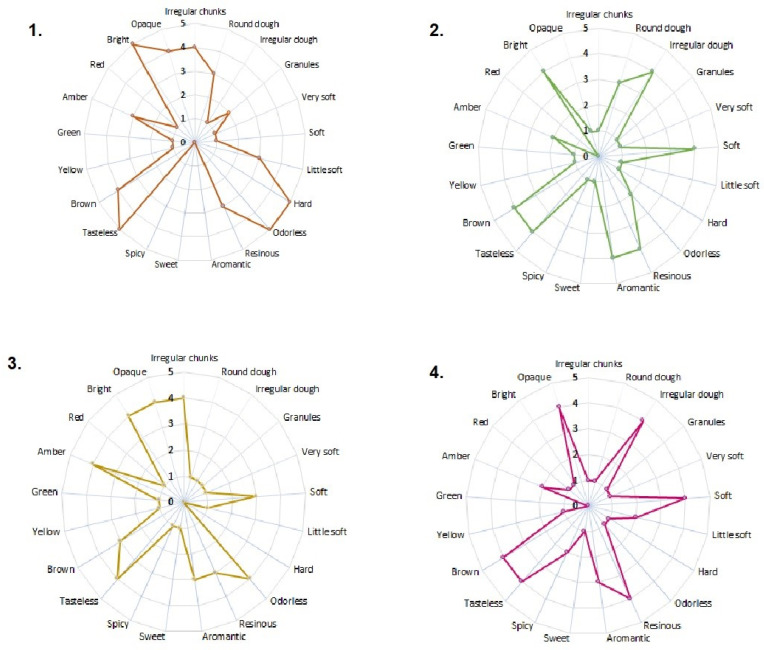
Sensory characteristics of raw propolis from (**1**) Algeciras, (**2**) Quebradon, (**3**) Otás, and (**4**) Arcadia from Huila, Colombia.

The sensory analysis results (color, aroma, consistency, and appearance) are presented in Figure 1. All samples were heterogeneous in their constitution and met the quality specifications established by the international regulations referenced in this study [23]. The samples from Algeciras and Otás presented an appearance of irregular pieces. At the same time, Quebradon and Arcadia are irregular masses, with data very similar to those reported by Viloria et al. [24] of propolis from lower Antioquia, Colombia, but different from the propolis from the region of Pasto [25], and although in all cases the collection method used was scraping [24,26], geographical and climatic conditions may probably be associated with these differences.

Regarding the consistency parameter, all the extracts were soft, unlike the one from Algeciras, which was rigid; it has been observed that this type of sample with a rigid appearance generally exhibits more excellent biological activity, whereas the soft or brittle ones, due to a high percentage of waxes and mechanical masses, may contain a low concentration of biologically active chemical compounds [24,27], making them less enjoyable from a pharmacological and commercial point of view.

In terms of consistency, many extracts were soft. However, the Algeciras extract was rigid, which is related to more excellent biological activity since propolis is malleable and brittle due to a high percentage of waxes and mechanical masses [25], reducing the number of active compounds [26], making them less attractive from a pharmacological and commercial point of view.

The aroma of the Algeciras extract was resinous, which is related to the properties of the wood of the plant from which the raw material was extracted. Other extracts were odorless, typical of propolis rich in essential oils [25,26]. All extracts were tasteless, a characteristic of propolis, composed of resins, phenols, and flavonoids.

The extracts varied from brown to amber in color due to the complex chemical composition of the constituents or the origin and profile of the pollen of the plant used for extraction [4,25], given that the flora and the wax content contribute to the sensory characteristics of propolis. Therefore, studying the pollen profile of Huila propolis is of great interest. In general, our results agree with those of other studies in which the same sensory characteristics were studied in Colombian propolis from other regions [24,25,26], as well as propolis from areas of Brazil [28,29], Portugal [4,30], Turkey [31], and Ethiopia [32], providing a future contribution to the standardization of propolis in Colombia.

### 2.2. Physicochemical Characteristics

Physicochemical parameters are shown in Table 1. Ethanolic propolis extracts (EEPs) yielded from the four samples were similar; however, EEPs from Arcadia had a lower yield (32.4 ± 0.9) than other propolis samples. By contrast, yields of propolis extracts from Indonesia [33], Morocco [34], Brazil [28], and other departments of Colombia (Nariño, Santander and San Andres, Antioquia) [26] had averages below Brazilian [23] and Argentine regulations (≥35%) [35], which can be attributed to various factors, such as vegetation, the period of obtaining the raw material, the beekeeping area, and species of producer bees [26,30].

Humidity is used to determine propolis quality [34]. The propolis samples studied exhibited similar humidity (Table 1). These values are below the regulations of Brazil (<8%) and Argentina (<10%), within the ranges reported for Morocco [34], Slovenia [36], and Colombia (Bajo Cauca) [24], and well below the values reported for Portugal [4], Brazil [28], and Colombia [25,26]. Humidity indicates handling and the environment in which propolis is produced. Our propolis samples showed a good percentage of humidity and a high concentration of bioactive compounds by weight, which may be associated with the biological activity of EEPs [24,37]. Low humidity prevents contamination of the extracts by environmental microorganisms and fungi, which produce toxins that affect human health [24].

Total ash content is a crucial parameter identifying adulteration in raw propolis [34,38]. In this study, the values of the ash content were similar in the samples (Table 1) and below the range of international regulations (<5.0%) [23], which indicated good quality and low degree of impurity. These data were similar for Brazilian [28], Moroccan [34], and Colombian propolis [24,25,26].

Wax content influences the quality of propolis. The percentages obtained in the EEPs of the samples (Table 1) studied were within the parameters required by international regulations (Brazilian < 25% and Argentina < 40%) and are similar to those described by Aboulghazi et al. for Moroccan propolis [34] and Figueiredo et al. for Brazilian propolis [28]. An increase in wax content can be related to the poor handling of raw materials during collection or propagation by bees mixing waxes with resins to fill the holes in the hive or traps [24,26,34].

Low melting point values were observed for the extracts (54–62 °C), being within the normative range for propolis (<100 °C), and the data were consistent with those obtained for propolis from Bajo Cauca in Colombia [24]. This parameter is related to the wax content and, in turn, the raw material’s texture. Correlating the melting points with the texture of the samples studied, we found little relationship between the melting point data and malleability [24,34].

**Figure 2:**
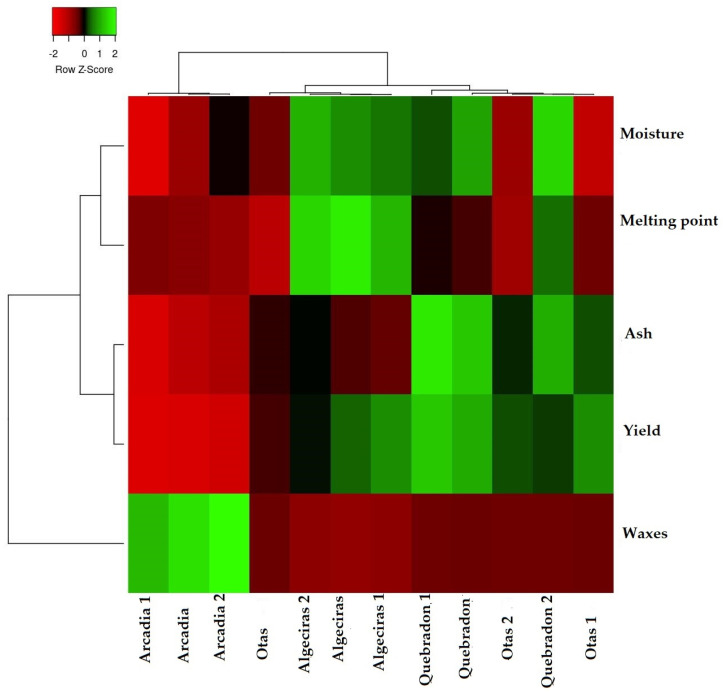
Cluster heat map of propolis samples and physicochemical parameters. The individual values of each propolis sample are represented as colors, with the highest data being red to black and the lowest data being dark green to light green.

To verify how the results of the physicochemical parameters allow us to classify and differentiate the four propolis evaluated, Figure 2 shows the heat map graph that shows the hierarchical structure of the cluster simultaneously as the propolis samples and the physicochemical variables (rows and columns, respectively), where three distribution groups are presented. The first group represents the direct relationship between humidity and melting point, where both variables are associated with the quality of the product and the process of propolis collection, handling, and production. The second group represents the variables determination of ashes and waxes, which are associated with the quality of propolis about the amount of impurities, plant material, and adulteration of the product that reduces the amount of bioactive compounds of the EEPs. The third group is the performance parameter directly related to the other two groups.

According to these results, the Quebradon sample presents the highest yield associated with the low amount of ashes and waxes. In contrast, Arcadia presented the lowest yield related to the high wax content. Notably, according to the physicochemical characteristics of propolis, this type of classification analysis allows an analysis of the quality of the products that can be used to develop drugs or phytotherapeutics.

Currently, only some studies report this type of analysis for propolis. However, as described by Dias et al., they are beneficial in samples of propolis of Portuguese origin, where it was demonstrated that humidity and pH are essential factors in their classification [4].

### 2.3. Chemical Characterization

**Figure 3:**
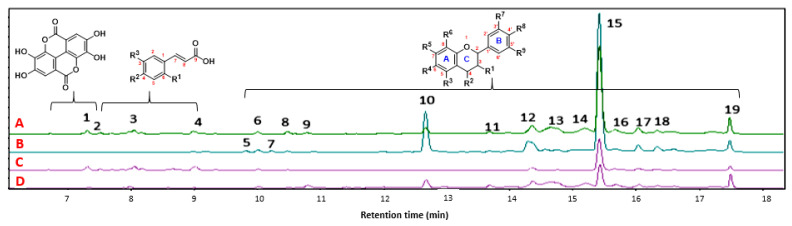
The chromatographic profile of the ethanolic extracts of propolis 1–4. ((**A**) Algeciras, (**B**) Quebradon, (**C**) Otás, and (**D**) Arcadia).

The four propolis samples were obtained from the Huila in different locations (altitude: 700–2000 m.a.s.l). The chemical characterization of the ethanolic extracts of propolis was performed using liquid chromatography coupled with mass spectrometry. In total, **19** characteristic signals were identified. The signal with a retention time of 15.5 min was most distinct. This signal was the majority signal in all extracts according to its relative abundance, and according to a review of databases, such as Knapsack and CHEMnetBASE-Dictionary of Natural Products, it is consistent with chrysoeriol-O-methyl-ether. The chromatographic profile is shown in Figure 3.

Chromatographic analysis of the four propolis samples obtained from different geographical locations in southern Colombia allowed for the identification of compounds **1**–**19** (Figure S1), which were identified by scrutinizing the details of their mass spectra using the precursor ion, fragmented ions, and comparison of the fragmentation patterns with the molecules described in the literature. Table 2 summarizes the identification of these compounds, and they are numbered according to their retention times. Compounds **1**–**4** (Figure S1, Table 2) were characterized as phenolic acids and flavonoids and have been reported in propolis samples obtained from various sources, such as resins of poplar, conifer, birch, pine, alder, willow, palm, *Baccharis dracunculifolia*, and *Dalbergia cataphyll*s [3].

On the contrary, it was possible to identify an essential group of secondary metabolites whose primary components are C6/C3/C6, corresponding to flavonoids (**5**–**19**), representing 79% of the chemical compounds of propolis considered in this study. Compounds **5** to **19** have also been described in propolis [39,40,41].

Notably, there was wide variability in the relative abundance of each compound (**1**–**19**) as a function of the corresponding propolis, which may be related to several factors, such as altitude, temperature, phytogeographic conditions, and the source of propolis (e.g., eucalyptus, oak, laurel, bees) [42,43]. The chemical composition and biological properties of propolis from different geographical origins have been the subject of numerous studies that have provided important information on the relationship between biological activity and chemical constituents, such as flavonoids [44,45].

Of the 19 elucidated compounds, it was found that for propolis extracts A, B, and D, the three significant compounds present are chrysoeriol-*O*-methyl-ether, chrysin-*O*-methyl-ether, and galangin. In contrast, for sample C, the most representative compounds are chrysoeriol-*O*-methyl-ether, ellagic acid, and 3,4-*O*-dimethylcaffeic acid, all of them also detected in Brazilian [28,46], Chilean [47], Portuguese [4], and European propolis [48,49]. They have ecological functions ranging from defense against microbial pathogens or herbivorous animals to protection from sunlight and can have simple or complex structures, as shown in fruits, vegetables, barks, roots, and leaves.

Flavonoid-type compounds and phenolic acids are among the most active in this resin, which acts in different physiological processes and performs various functions, including antimicrobial and antioxidants. Studies on the chemical composition of propolis can help establish criteria for quality control of the samples using standardized parameters as described by Brazilian regulations. The classification of the chemical composition of Colombian propolis is crucial to improve our knowledge of the chemistry of this product and its directly related biological properties.

### 2.4. Total Phenolic Content, Total Flavonoid Content, and DPPH• Radical Scavenging Capacity

The content of phenols and flavonoids in propolis is an important parameter that establishes both the quality of the material and its biological potential, especially antioxidants. Total phenolic content (TPC) for propolis extracts ranged from 221 ± 4.1 to 543.1 ± 8.4 mg GAE/g DP. Propolis extracts B (Quebradon) and D (Arcadia) had the highest phenolic content: 255.9 ± 9.2 and 543.1 ± 8.4 mg GAE/g DP and the best antioxidant capacity in the 2,2-diphenyl-1-picryhydrazyl (DPPH) test, with mean inhibition values of 37.42 ± 1.2 and 38.19 ± 1.2 µg/mL (*p* < 0.05), respectively, which were comparable with the commercial standard antioxidant BHT (IC_50_ = 32.1 ± 2.4 µg/mL) (Table 3). The results obtained are of great importance because they describe the antioxidant properties of propolis as a function of phenolic compounds and flavonoid contents. These results can be explained by a higher relative abundance of compounds **10**, **12**, **15**, and **19** in propolis B and D (Figure 3), whose antioxidant properties and potential to reduce oxidative stress are described [50,51,52].

Total flavonoid content (TFC) was expressed as milligrams of quercetin equivalents per gram of propolis extract (mg QE/g PE). Propolis extracts from Quebradon and Arcadia showed the best results, with TFC > 30 mg QE/g PE compared with the standard (Table 3). These data are consistent with the chromatographic profile in Figure 3, in which we see structural blocks of flavonoids corresponding to compounds **10** and **15**, whose relative abundance is the highest in Quebradon and Arcadia extracts. There is an essential contribution to the flavonoid content due to compounds **12**–**13** and **16**–**19**, which also correspond to flavonoids with more moderate relative abundances. Finally, extracts from Algeciras and Otás had the lowest flavonoid content (TFC < 6 mg QE/g PE), given the low relative abundance or absence of many of these compounds in the chromatographic profile (Figure 3). Notably, these data are consistent with the results of TPC and antioxidant activity, which were the lowest for these two extracts.

Likewise, the values of phenols and flavonoids found in propolis from the Huila region are higher than those reported in other regions of Colombia, as described by Salamanca et al. [26] and Palomino et al. [53]. Additionally, the extracts from Quebradon and Arcadia presented contents of phenolic compounds and flavonoids similar to or higher than those reported for propolis of European, Asian, Portuguese, and South American origin [54], which suggests that the composition of the propolis analyzed is similar to that found in temperate regions, due to the influence and diversity of the botanical origin from which the bees make them in each of the biogeographic zones.

Regulations such as those of Argentina [35] and Brazil [23] have established minimum concentration requirements that propolis and its extracts must meet to be used as raw materials in the development of products; for phenolic compounds, the minimum value is 50 mg GAE/g EEP, and for flavonoids, 5 mg QE/g EEP [35]. By the above, the evaluated propolis meets the quality requirements established by these regulations.

### 2.5. Biological Activity

#### 2.5.1. Effects of EEPs on Cell Viability and Determination of IC_50_

**Figure 4:**
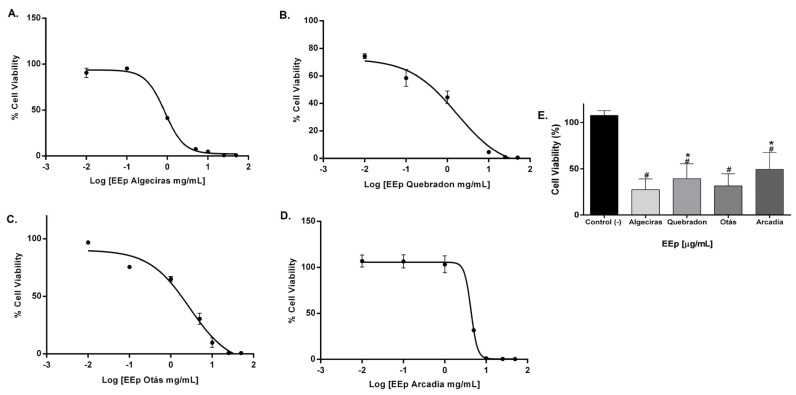
Dose–response curves of the effect on the viability of EEP cells from propolis samples in the GHnF cell line. Cells were treated for 48 h with different concentrations (0.01–50 mg/mL). (**A**) Algeciras, (**B**) Quebradon, (**C**) Otás, (**D**) Arcadia, (**E**) comparison of the IC_50_ of the extracts in the HGnF cell line. The results are the average of three independent tests with three repetitions (*n* = 3) ± standard error of the mean (SME). (#) represents the significant differences in the IC_50_ of each extract compared to the control group (untreated cells), and (*) represents the significant differences comparing the IC_50_ between the propolis samples.

The effect of EEPs on the cell viability of L929 and HGnF cells was evaluated for 48 h using the Alamar Blue method. EEPs from Algeciras had the most significant cytotoxic effect on the human fibroblast cell line with an IC_50_ of 0.8681 mg/mL, being extremely cytotoxic at concentrations of 1.0–50 mg/mL according to the ISO 10993 Standard [55]. In contrast, the safest extract was that of Arcadia, with an IC_50_ of 4.288 ± 1.4 mg/mL (*p* < 0.05). The extracts of Quebradon and Otas (moderately cytotoxic) had an IC_50_ of 2.833 ± 2.3 (*p* < 0.05) and 1.701 ± 1.4 mg/mL, respectively (Table 4), decreasing cell viability in the range of 1.0–50 mg/mL (Figure 4).

**Figure 5:**
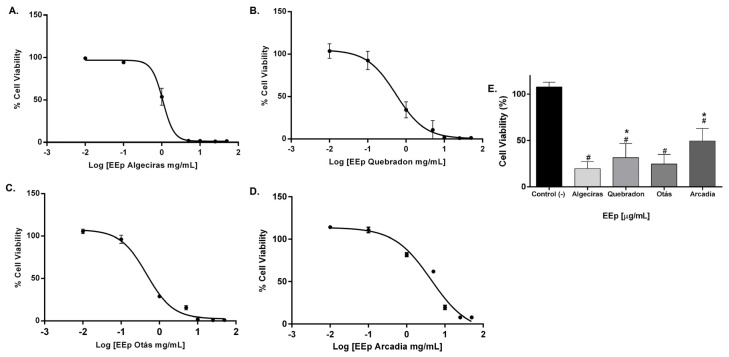
Dose–response curves of the effect on the viability of EEP cells from propolis samples in the L929 cell line. The cells were treated for 48 h with different concentrations (0.01–50 mg/mL). (**A**) Algeciras, (**B**) Quebradon, (**C**) Otás, (**D**) Arcadia. (**E**) Comparison of the IC_50_ of the extracts in the L929 cell line. The results are the average of three independent tests with three repetitions (*n* = 3) ± standard error of the mean (SME). (#) represents the significant differences in the IC_50_ of each extract compared to the control group (untreated cells) and (*) represents the significant differences comparing the IC_50_ between the propolis samples.

The level of toxicity in the murine gingival fibroblast cell line (L929) was similar to that in human gingival fibroblasts, and the propolis extract from Otás had higher cytotoxicity, with an IC_50_ of 0.3206 ± 4.16 mg/mL. In contrast, the safer extract was the same as in HGnF cells, Arcadia, with an IC_50_ of 4.34 ± 11.9 mg/mL (Table 5). By contrast, EEP samples from Quebradon and Algeciras had IC_50_ values of 2.578 ± 8.0 and 0.5959 ± 1.17 mg/mL, respectively (*p* < 0.05) (Figure 5), inducing moderate cytotoxicity in both fibroblast cell lines.

The cytotoxic effects of different types of propolis extracts demonstrate different effects according to the sensitivity of the cells. Propolis has low toxicity in healthy or normal cells [46,56]. In this study, Arcadia and Quebradon extracts had low cytotoxicity toward human and mouse fibroblasts, exerting their effects at >1000 mg/mL. These findings are consistent with the effects of Brazilian green propolis [57] on gingival and periodontal fibroblasts or red and brown propolis on pulpal ligament cells and pulpal fibroblasts [58]. Propolis extract can act as a storage solution to maintain the viability of pulpal ligament cells in avulsed teeth.

L929 cells have been widely used to evaluate the cytotoxic effects of biomaterials or natural products and are recommended by international standards for their sensitivity [43]. The results of this study demonstrate low toxicity of Huila propolis extracts by inducing cytotoxic effects at >500 mg/mL, compared with other studies where yellow or red propolis induced cytotoxic effects in an IC_50_ range of 17–18 µg/mL, presenting greater sensitivity compared with other standard cell lines such as fibroblasts, astrocytes, or epithelial cells [43].

Although we only studied the effects of EEPs on normal cells to determine the safety profile and dose that must be evaluated in future studies on antimicrobial, anti-inflammatory, or regeneration activity in the oral cavity, propolis can have more significant cytotoxic effects on tumor cells compared with normal cells [43]. The high selectivity of propolis in colon cancer cells, prostate, HeLa, and leukemia cells compared with epithelial cells, fibroblasts, and lung cells (V-79) can be correlated with the higher proliferation index of tumor cells [43,59,60]. Similarly, the difference in results obtained from the cytotoxic effects of propolis in healthy and tumor cells is related to the content of active chemical compounds, environmental and climatic factors, the extraction process, and cellular sensitivity. Therefore, it is necessary to continue evaluating the cytotoxic profile of propolis and its active compounds to establish analytical standards.

Of the chemical components present in propolis, it has been shown that flavonoids such as galangin, quercetin, and chrysin, identified in propolis samples from the Huila region, have anticancer, antitumor, and cytotoxic effects on various cells by inhibiting DNA synthesis, the induction of apoptosis through various mechanisms associated with proapoptotic pathways such as BA, BID, Bax, caspases 3, 8, and tumor suppressors including p53, cell cycle inhibitors such as some cyclin-dependent kinases, and ceramide activator cascades and their messengers in tumor cells [61]. However, little is known about the mechanisms of cytotoxicity induced by chrysin.

As demonstrated in this study, propolis samples, mainly from Otás and Quebradon, presented cytoprotective effects on healthy cells, already described in other types of propolis, considering that it could be related more to a synergism than to the effect of one or more compounds [62]. However, various studies have shown that the significant flavonoid compounds in propolis extracts have protective effects on healthy neural, liver, kidney, and immune cells through mechanisms that block oxidative stress and the activation of caspases, inhibiting apoptosis [63,64].

These differences between the effects of flavonoids and propolis are of great interest due to their impact on the therapy of various diseases; however, new studies are required to support or expand information on the mechanisms and synergy that may occur between the different compounds present in propolis.

#### 2.5.2. Effect of Propolis on Fibroblast Proliferation

**Figure 6:**
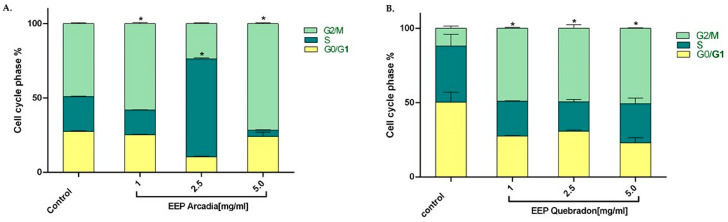
Effect of propolis extracts from Arcadia (**A**) and Quebradon (**B**) on the cell cycle in HGnF cells. Cells were treated at 1.0, 2.5, and 5.0 mg/mL extract for 48 h. The results are the average of three independent tests in duplicate (*n* = 6) ± SME. The effects of the extracts were compared with the control (untreated cells) * *p <* 0.05.

We examined the effect of the two safest extracts, Arcadia and Quebradon, on the cell cycle in HGnF cells to develop safe and pharmacologically beneficial phytotherapeutics for periodontal and endodontic disease. Flow cytometry analysis of the distribution of the cell cycle after 48 h of exposure to propolis extract showed that Arcadia EEP (1.0–5.0 mg/mL) increased the percentage of cells in the G2/M phase (49.1% and 71.6%, respectively); however, at 2.5 mg/mL, the cells were distributed in the S phase (65%), compared with the control group (untreated cells) (*p* < 0.05) (Figure 6A), which were 49.1% in the G2/M phase. By contrast, the Arcadia EEP increased the percentage of HGnF cells in the G2/M phase at the concentrations evaluated (1.0 mg/mL = 49.1%, 2.5 mg/mL = 49.5%, and 5.0 mg/mL = 50.8%) compared with the control group (11.9%). These results suggest that Arcadia and Quebradon propolis induce proliferative effects associated with an increase in the cell cycle in the G2/M phase (Figure 6B).

Thus, propolis EEPs can induce proliferative effects, increasing the percentage of cells in the G2/M phase of the cell cycle, which is of great therapeutic interest in cell cycle regulation and the anti-inflammatory response in periodontitis. Microorganisms such as *Porphyromonas gingivalis* can modify the cell cycle in gingival fibroblasts or primary periodontal ligament cells, causing G1 arrest and suppressing cell proliferation and apoptosis [65,66]. However, we found that the Arcadia EEP has a biphasic hormetic-type effect [67,68], which has been described for natural compounds and other extracts of Turkish propolis in periodontal ligament fibroblasts and epithelial cell lineage of ductal carcinoma (UACC) [69] and Polish propolis in L929 cells, human basal alveolar epithelium cells (A549), and lung cancer epithelial cells (H23) [68]. This phenomenon correlates with different and even opposite results of a compound or extracts at different concentrations or doses of treatment. This phenomenon is believed to be due to compound agonism, where both activate or inhibit signaling pathways [67,68], with a greater capacity to inhibit low concentrations but a greater affinity and activation at high concentrations. However, in fibroblasts, this phenomenon cannot be easily explained; therefore, additional studies on Huila propolis must be performed to elucidate the mechanisms involved in activation or cell cycle arrest.

The antiproliferative effects of propolis and its most representative compounds, such as flavonoids (apigenin, quercetin, luteolin, and chrysin), have been previously described [64,70,71]. Chrysin demonstrated that its effects are mediated by cell cycle arrest in G1, associated with an increase in the expression of the p21 inhibitor and the reduction of the cyclin kinase, p21 (Waf1/Cip1) [64,70]. On the other hand, it has been shown that quercetin, genistein, and chrysin can have cytotoxic effects in different types of cancer cells by arresting the G(2)/M cell cycle through the positive regulation of p21 (waf1) and the negative regulation of cyclin B1 in both the mRNA and the protein [64,70,71]. Similarly, cell cycle arrest effects have been demonstrated in dermal and gingivalis fibroblasts in the G2/M phase without an increase in proliferation [64,70,71]. These data are very similar to those obtained in this study with the Quebradon and Arcadia samples, which may result from the antiproliferative effect of the tested extracts, so new studies are required to determine the effect of this propolis on cell proliferation and cell replication, such as control points in normal cells, keeping in mind the great medical importance and pharmacological properties of propolis and its compounds, mainly flavonoids, for the treatment of some common diseases.

In comparison with Colombian propolis from the Cauca and Cundinamarca region, our results differ from those reported by Torres et al. [72], where these extracts presented cytotoxic effects and, in turn, induced retention of the cell cycle in the G0/G1 phase in canine fibroblasts. This may be associated with cellular sensitivity or a chemical composition rich in benzophenes and diterpenes unlike the propolis of the Huila, evaluated in this study, which demonstrates the great variety and different effects that this natural product can present.

## 3. Materials and Methods

### 3.1. Obtaining Propolis and Preparing Ethanolic Extracts

Propolis **1**–**4** were obtained from four regions of the Huila-Colombia department (southern region). Propolis A was obtained from the municipality of Algeciras (2°31′19″ N 75°18′52″ W), propolis B from the Quebradon municipality of Algeciras (2°33′55″ N 75°15′22″ W), propolis C from Otás-Campoalegre Huila (2°36′10″ N 75°20′05″ W), and propolis D from Arcadia-Algeciras Huila (2°31′19″ N 75°18′52″ W). Sampling was carried out using scraping directly from the inside of the hives, removing the product adhered to the lateral faces.

Approximately 150 g of propolis was weighed and ground with a mortar, cold macerated with 96% ethanol for 72 h, and filtered through a funnel with a paper filter (Whatman no. 4) to remove particles. The excess solvents were eliminated by distillation under reduced pressure subjected to rotary evaporation (IKA RV 10auto pro, Staufen, Germany) at 40 °C. Three clarifications were performed to obtain a higher yield and excellent extraction process. Finally, EEPs A–D were obtained, stored in a desiccator to remove solvent residues, and used for physicochemical characterization, chromatographic analysis, quantitative assays of phenolics, flavonoids, and DPPH, and evaluation of cytotoxicity and the effect on the cell cycle in fibroblast cell lines. Performance was calculated using the following equation [73]:

$$Yield\left(\%\right)=\frac{W\;propolis\;extract\;residue}{W\;initial\;dried\;propolis}\times 100$$

### 3.2. Sensorial Analysis

Samples of raw propolis material were used to evaluate the sensory characteristics according to the Ministry of Agriculture of Brazil (Normative Instruction No. 11, of 20 October 2000) [23] since there is no legislation regarding the quality control of propolis in Colombia. Sensory analysis was performed as described by Lozina et al. [27] and Virola et al. [24], with some modifications.

Sensory parameters (appearance, consistency, color, and taste) were determined by descriptive sensory tests carried out by a panel of 10 semi-trained people who rated the propolis samples using interval scales. Five-level scales were used as follows: appearance: round dough (1), irregular dough (2), irregular opaque pieces (3), irregular chunks (4), and granules (5); odor: odorless (1), resinous (2), aromantic (3), aromatic resinous (4), and very aromatic resinous (5). To evaluate the consistency and flavor attributes, a four-level scale was used: consistency: very soft (1), soft (2), slightly soft (3), and complex (4); flavor: spicy (1), sweet (2), bitter (3), and tasteless (4). Appearance and color were determined by visual observation and consistency by pressing the sample with the fingers.

### 3.3. Physicochemical Characteristics

#### 3.3.1. Moisture

The determination of moisture was ascertained by the thermogravimetric method. Approximately 1.0 g of raw material was weighed in a tared porcelain capsule and placed in an oven at 105 °C for 3 h. Subsequently, it was allowed to cool in a desiccator until a constant weight was obtained (two successive measurements should not differ by more than ±5 mg). The percentage of loss due to heating was calculated using the following equation:

$$Moisture\left(\%\right)=\frac{100\times [A1-A2]}{A2}$$

where *A*1 = sample weight; *A*2 = weight of dry sample.

#### 3.3.2. Determination of Melting Point

The melting point was determined using the capillary method described by Viloria et al. [24]. Approximately 10 mg of raw propolis was cold-macerated with liquid nitrogen until the desired particle size was obtained. Subsequently, it was placed inside a capillary, and the temperature of digital fusiometer (Electrothermal, St. Louis, MO, USA) was raised until the state change was evident, recording the temperature value.

#### 3.3.3. Ash Content

Ash determination was performed through the calcination method [23,24]. In total, 1.0 mg of raw propolis was taken in a tared and water-free crucible, calcinated in a muffle at 550 °C for 3 h, and desiccated until a constant weight was obtained. Ash was calcinated using the following equation:

$$Ash\;\left(\%\right)=[\frac{m1-m2}{m0}]\times 100$$

where *m*1 = mass of the capsule and ash, *m*2 = mass of the capsule before calcination, and *m*0 = mass of propolis.

#### 3.3.4. Determination of Waxes

The gravimetry method was used to determine the amount of wax in propolis samples, according to Viloria et al. [24], and the protocols of Brazilian regulations [23] were implemented. The crude material of each propolis sample (10 mg) was placed in a cellulase thimble. Soxhlet extraction was performed for 6 h with 96% *v*/*v* ethanol. Precipitated wax was removed by filtration through a Whatman No. 1 filter paper. The waxy precipitate retained on the paper was dried in an oven at 45 °C until dry, and a constant weight was maintained. The percentage of wax was expressed as weight difference (*w*/*w*).

### 3.4. Chemical Characterization

#### High-Performance Liquid Chromatography Coupled with Mass Spectrometry Analysis

High-performance liquid chromatography coupled with mass spectrometry analysis. High-resolution mass spectrometry analyses with electrospray ionization (ESI) were performed using a Bruker (Billerica, MA, USA) micrOTOF–QII mass spectrometer coupled to a Shimadzu (Kyoto, Japan) Prominence liquid chromatography system, consisting of two analytical pumps: model LC-20AD (Shimadzu, Kyoto, Japan), with a SIL-20AHT automatic injector, SPD-20A UV/Vis detector, CTO-20A column oven, and CBM-20A controller. Each propolis analyzed by this technique was prepared at 1 mg/mL using LCMS-grade methanol. The column used was a Phenomenex (Torrance, CA, USA) Luna C18 (5 µm, 150 mm × 2 mm). The flow was 0.2 mL/min, and the mobile phase was a mixture of solvents A (0.1% HCOOH in H_2_O) and B (0.1% formic acid in MeOH). The gradient started at 5% B (0 min) and was maintained for 2 min. Then, B was incremented to 100% from 5 to 30 min and maintained for 5 min. The oven temperature was 40 °C, and the wavelength was 254 and 280 nm. The ESI interface was operated in positive ion mode with 4.5 kV in the capillary and 0.5 kV in the endplate offset. The pressure of the nebulization gas was 0.4 Bar; the drying gas was maintained at a flow rate of 8 L/min at 200 °C. The collision and the quadrupole energy were set to 12 and 6 eV, respectively. RF1 and RF2 funnels were programmed to 150 and 200 Vpp, respectively. The mass spectra were calibrated using sodium formate.

The molecular formulas of the compounds were determined using mass measurements obtained from low-resolution spectra. When possible, the chemical nature of the compounds was determined using the databases Knapsack (http://ka-naya.naist.jp, accessed on 18 March 2021) and CHEMnetBASE-Dictionary of Natural Products (http://dnp.chemnetbase.com, accessed on 18 March 2021).

### 3.5. Determination of Bioactive Compounds

#### 3.5.1. DPPH^•^ Free Radical Scavenging Activity Measurements

Free radical scavenging activity using DPPH^•^ was determined following a standardized method [74]. Ethanolic solutions with variable concentrations (0–500 ppm) of crude ethanolic extracts were prepared: 10 mM DPPH^•^ (190 μL) was separately added to each ethanolic extract solution (10 μL). This mixture was incubated at room temperature for 1 h in the dark, and absorbance at 515 nm was measured. Three replicates were evaluated for each determination. BHT was used as a positive control. DPPH value was expressed as half-maximal inhibitory concentration (IC_50_) and calculated using GraphPad Prism 7.0 software.

#### 3.5.2. Total Phenol Content (TPC) and Total Flavonoid Content (TFC)

TPC was determined using Folin–Ciocalteu reagent and 7.35% Na_2_CO_3_ solution [75]. For TPC, a set of EEPs with initial concentrations (0–500 ppm) adjusted to absorbance at 765 nm, fell in the range of 0.08–0.8 absorbance units. To 20 μL of the adjusted solution, 10% Folin–Ciocalteu reagent (40 μL) and 7.35% sodium carbonate (150 μL) were added. The mixture was incubated in the dark at room temperature for 2 h, and absorbance was measured at 765 nm. Absorbance values were interpolated into a standardized calibration curve (absorbance vs. gallic acid concentration). All tests were performed in triplicate. The results were expressed in mg GAE/g DP (mg gallic acid equivalent per gram dry propolis).

The TFC was determined using the method of Kumazawa et al. [30]; 70 μL of ethanolic extract solution was incubated with a mixture comprising ethanol (50 μL), 10% aluminum trichloride (10 μL), and 0.1 M sodium acetate (10 μL) for 40 min in the dark. Absorbance values measured at 420 nm were interpolated into a standard calibration curve (absorbance vs. quercetin concentration). All tests were performed in triplicate. TFC values were expressed as milligrams of quercetin equivalents per gram of dry propolis (mg QE/g DP).

### 3.6. Biological Activity Assays

#### 3.6.1. Cell Lines and Culture Conditions

Cell cultures of the suckling mouse skin cell line L929 (ATCC CRL-6364) and gingival fibroblast cell line isolated from human gingiva (HGnF 2620, Sciencell, Carlsbad, CA, USA) were used. Cultures were maintained in Dulbecco’s Modified Eagle Medium (DMEM), supplemented with 10% fetal bovine serum (Hyclone, Logan, UT, USA) penicillin–streptomycin (100 IU; 100 μg/mL) in a humidified atmosphere of 5% CO_2_ at 37 °C.

#### 3.6.2. Cell Viability Assay and IC_50_ Determination

The Alamar Blue assay (Biosource, Camarillo, CA, USA) was used to determine the effect of propolis samples on cell viability. It was used through fluorometric detection after the reduction of resazurin in the resorufin product [76]. L929 and HGnF cells were seeded in 96-well plates at a concentration of 5 × 10^3^ cells/well in 100 μL/well of DMEM. After the stabilization period (24 h), the medium was removed, and the cells were treated for 48 h with 0.01, 0.1, 1.5, 10, 25, and 50 mg/mL propolis extract (prepared as stock solutions of 100 mg/mL in 1% dimethyl sulfoxide Sigma-Aldrich, St. Louis, MO, USA, #Q3251). The extracts were removed. The cells were washed with PBS and incubated at 37 °C for 4 h with 100 μL of 0.44 μM resazurin solution prepared in culture medium. Fluorescence was measured using a plate reader (TECAN Infinite 200 PRO, Mennedorf, Switzerland) at 570 nm with a 630 nm differential filter.

Fluorescence values were transformed into percentages of cell viability compared with untreated cells and plotted as a function of the logarithm of the treatment concentration. IC_50_ was calculated from a linear regression between cell viability, and extract concentration [77] was determined using GraphPad Prism 7.0 software (GraphPad Corp., San Diego, CA, USA). Three independent experiments were performed in triplicate.

The level of toxicity was determined based on the ISO 10993 Standard, which highlights the classification of cytotoxicity based on the percentage of cell viability: 100–75%: non-cytotoxic; 74–50%: slightly cytotoxic; 49–25%: moderately cytotoxic; and 24–0%: extremely cytotoxic.

#### 3.6.3. Cell Cycle Analysis

To determine if propolis extracts can induce changes in DNA content in different phases of the cell cycle, increasing the proliferation of HGnF cells, the 7-amino actinomycin D (7-AAD) [78] method was used. Thus, 2 × 10^4^ cells/well were seeded in 24-well plates. After 24 h of culture stabilization, the cells were treated with propolis extracts, demonstrating lower toxicity levels at concentrations of 1.0, 2.5, and 5.0 µg/mL for 48 h. The culture medium was removed, and the cells were dissociated with trypsin–EDTA solution (0.25–0.5 mM) for 5 min at 37 °C and washed twice with PBS. The cell pellet was fixed with 70% ethanol and stained with 25 mg/mL 7-AAD solution (BD Pharmingen, San Diego, CA, USA) for 15 min at 20–25 °C in the dark. Cell cycle analysis was performed by flow cytometry on the BD Accuri C6 device using a 488–647 nm laser. The results were analyzed using Modfit LT software version 5.0 and graphed using GraphPad Prism 7.0 software. Unstimulated cells were used as negative controls. For this study, three independent experiments were performed in duplicate.

### 3.7. Statistical Analysis

The cluster heat map was created using R software (R Development Core Team, Vienna, Austria, 2012). All experiments were performed in triplicate for at least three independent experiments, and results were expressed as means ± the standard error of the mean (SME). The data were subjected to analysis of variance (ANOVA) and Tukey’s multiple comparison test using GraphPad Prism 7.0 software. The level of significance was set at *p* < 0.05.

## 4. Conclusions

This study demonstrates, for the first time, new knowledge about the characteristics and factors to be considered as criteria for evaluating the quality of Colombian propolis from the Huila region. The physicochemical, sensory characterization, and antioxidant activity analyses of the four propolis comply with Brazilian legislation, advancing quality processes in Colombian propolis. Likewise, 19 compounds characterized as phenolic acids and flavonoids were identified, the most representative being chrysoeriol-*O*-methyl-ether, chrysin-*O*-methyl-ether, and galangin, which may be responsible for the antioxidant effects identified in this study. Among the biological effects, Arcadia and Quebradon propolis presented low levels of cytotoxicity with IC_50_ values more excellent than 2.0 g and detection of the cell cycle in gingival and L929 fibroblasts in the G2/M phase, which may be related to a cytoprotective, proliferative, or regenerative effect. Although new studies are required to identify the mechanisms in healthy cells clearly, the results of this study provide the basis for quality and biological effects to produce propolis with therapeutic and cosmetic potential.

## Figures and Tables

**Table 1:** Physicochemical characteristics of the propolis samples. Brazilian normative reference values [23].

	Algeciras	Quebradon	Otás	Arcadia	Reference Values
**Moisture** **(%)**	2.8 ± 0.4 *	2.1 ± 0.9	1.6 ± 06	1.7 ± 0.3	<10%
**Melting** **point (°C)**	59–61 °C *	59–62 °C *	55–57 °C	54–58 °C	60–100 °C
**Ash** **(%)**	2.14 ± 0.5	2.19 ± 0.1 *	1.98 ± 0.7	1.40 ± 0.6	<5.0%
**Waxes** **(%)**	6.6 ± 0.7	7.8 ± 0.3	7.9 ± 0.8	17.9 ± 2.1 *	<40%
**Yield** **(%)**	39.5 ± 1.2	48.5 ± 1.7 *	40.9 ± 1.1	32.4 ± 0.9	-

Results are presented as mean ± SEM of three independent experiments in duplicate. * (*p* < 0.05).

**Table 2:** Primary chemical constituents of propolis extract are annotated by liquid chromatography coupled with high-resolution mass spectrometry with electrospray ionization (LC-ESI-HRMS).

Peak	^a^ rt (min)	RA ^b^				*m*/*z* ^c^ ([M + H]^+^)	Molecular Formula ^d^	Error (ppm) ^e^	Name ^g^
		Propolis A	Propolis B	Propolis C	Propolis D				
1	7.3	7.1	0.2	2.7	1.4	300.9972	C_14_H_6_O_8_	4.14	ellagic acid
2	7.5	1.9	0.0	1.0	0.0	163.0388	C_9_H_8_O_3_	4.42	*p*-coumaric acid
3	8.1	5.9	0.1	1.2	1.7	193.0511	C_10_H_10_O_4_	−5.26	ferulic acid
4	9.0	7.6	0.3	2.9	1.0	207.0649	C_11_H_12_O_4_	4.03	3,4-*O*-dimethylcaffeic acid
5	9.8	0.0	0.5	0.1	0.0	315.0512	C_16_H_12_O_7_	−2.29	quercetin-*O*-methyl-ether
6	10.1	2.3	0.9	1.8	2.5	271.0613	C_15_H_12_O_5_	−2.40	pinobanksine
7	10.3	0.0	0.4	0.0	0.0	283.0959	C_17_H_16_O_4_	4.01	pinocembrin-*O*-methyl-ether
8	10.5	1.0	0.5	1.6	1.1	269.0462	C_15_H_10_O_5_	−4.46	apigenine
9	10.8	0.9	0.1	1.2	4.7	285.0391	C_15_H_10_O_6_	2.86	kaempferol
10	12.7	0.0	16.8	3.9	11.7	267.0648	C_16_H_12_O_4_	3.50	chrysin-*O*-methyl-ether
11	13.8	0.9	0.4	1.4	4.1	315.0512	C_16_H_12_O_7_	−2.29	rhamnetine
12	14.4	4.8	8.3	6.2	9.0	269.0461	C_15_H_10_O_5_	−4.09	galangine
13	14.8	0.6	0.6	1.1	1.0	283.0614	C_16_H_12_O_5_	−2.65	acacetine
14	15.2	0.0	1.3	4.8	8.3	329.0672	C_17_H_14_O_7_	−3.25	quercetin-*O*-dimethyl-ether
15	15.4	55.5	59.8	55.9	30.4	313.0703	C_17_H_14_O_6_	2.92	chrysoeriol-*O*-methyl-ether
16	15.7	1.5	1.4	2.8	4.3	327.0855	C_18_H_16_O_6_	4.17	pinobanksin-3-*O*-propionate
17	16.0	3.1	2.9	3.5	4.2	373.1643	C_21_H_26_O_6_	2.18	pinobanksin-3-*O*-pentanoate
18	16.3	1.9	1.8	1.3	2.1	341.1033	C_19_H_18_O_6_	−2.30	pinobanksin-3-*O*-butyrate
19	17.5	5.2	3.7	6.6	12.6	353.1015	C_20_H_18_O_6_	2.87	pinobansin-3-*O*-pentenoate

^a^ rt = retention time; ^b^ RA = relative abundance from normalized peak intensity in total ion current chromatograms; ^c^ accurate mass of the quasi-molecular ion ([M + H]^+^); ^d^ molecular formula deduced from accurate mass measurements; ^e^ calculated from the respective monoisotopic mass; ^g^ identified compounds based on diagnostic evidence, phylogenetic filtering, and database and literature data comparison.

**Table 3:** Content of total phenols, total flavonoids, and antioxidant activity of ethanolic extracts of propolis.

Propolis (EEP)	TPC (mg GAE/g)	TFC (mg QE/g)	DPPH (µmol/g)
Algeciras	221 ± 4.1	5.1 ± 0.26	23.4 ± 4.2
Quebradon	255.9 ± 9.2	31.2 ± 0.91	37.42 ± 1.2 *
Otás	234.4 ± 4.3	5.8 ± 0.71	29.53 ± 3.0
Arcadia	543.1 ± 8.4 *	32.5 ± 1.18	38.19 ± 0.7 *
BTH	--	--	32.1 ± 2.4
Q	--	61 ± 0.11	--

TPC = total polyphenol content, TFC = total flavonoid content, Q = quercetin, -- = Not rated. Values represent the means of three independent experiments ± the mean standard error (SEM), (* *p* < 0.05).

**Table 4:** IC_50_ values for each of the varieties of ethanolic propolis extract in the HGnF cell line. * *p* < 0.05.

Propolis (EEP)	IC_50_ Range (mg/mL)	IC_50_ (mg/mL)	R Square	SME
Algeciras	0.745–1.011	0.8681	0.98	±1.5
Quebradon	1.863–4.307	2.833 *	0.92	±2.3
Otás	0.7558–3.830	1.071	0.95	±1.4
Arcadia	2.579–7.130	4.288 *	0.94	±1.4

**Table 5:** IC_50_ values calculated for each variety of ethanolic propolis extract in the L929 cell line. * *p* < 0.05.

Propolis (EEP)	IC_50_ Range (mg/mL)	IC_50_ (mg/mL)	R Square	SME
Algeciras	0.4955–0.7175	0.5959	0.99	±1.17
Quebradon	1.484–4.480	2.578 *	0.94	±8.0
Otás	0.1688–0.6090	0.3206	0.92	±4.16
Arcadia	2.415–7.809	4.341 *	0.99	±11.9

## Data Availability

The data supporting the findings of this study are available from the corresponding author upon reasonable request.

## Supplementary Materials

The following supporting information can be downloaded at: https://www.mdpi.com/article/10.3390/molecules29071643/s1, Figure S1: compounds **1**–**19** found in Colombian propolis extracts.
