# Supplementary material for: Physicochemical Characterization, Antioxidant, and Proliferative Activity of Colombian Propolis Extracts: A Comparative Study
Source: Molecules. 2024 Apr 6;29(7):1643. doi: 10.3390/molecules29071643 (PMC11013913; doi:10.3390/molecules29071643)
Supplement: Supplementary file 1 [file molecules-29-01643-s001.zip › molecules-2827446-supplementary.pdf]

Article

Physicochemical Characterization, Antioxidant, and Proliferative Activity of Colombian Propolis Extracts: A Comparative Study

Diana Marcela Buitrago [1]*, Sandra J. Perdomo [2], Francisco Arturo Silva [3], Willy Cely-Veloza [4, 5], Gloria Inés Lafaurie [1]*

Supplementary Material

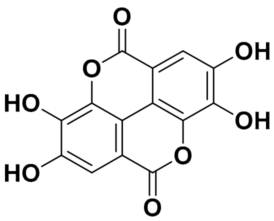

(1)

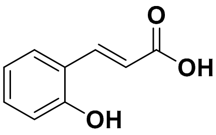

(2)

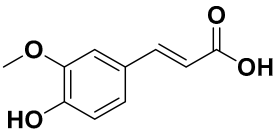

(3)

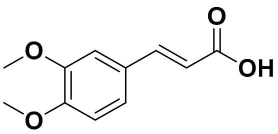

(4)

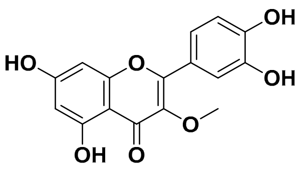

(5)

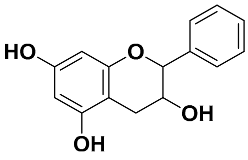

(6)

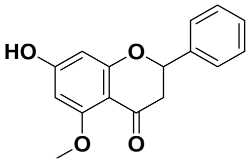

(7)

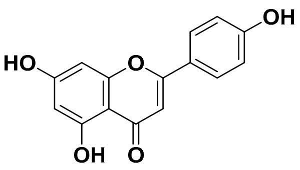

(8)

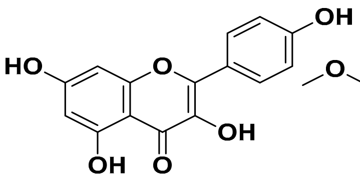

(9)

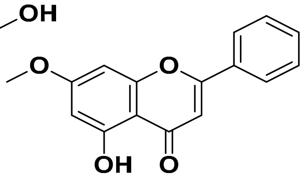

(10)

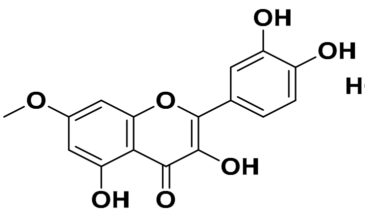

(11)

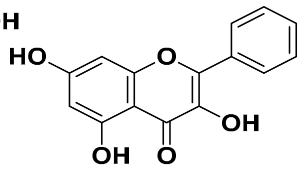

(12)

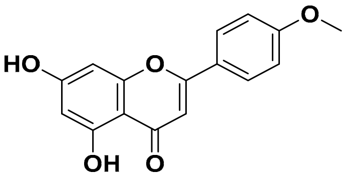

(13)

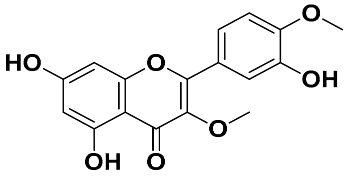

(14)

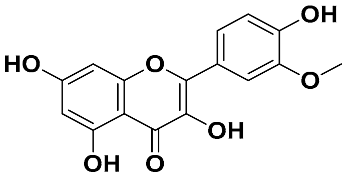

(15)

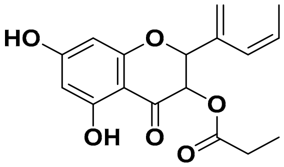

(16)

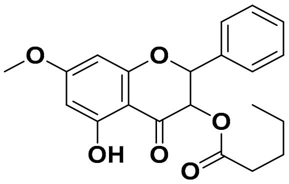

(17)

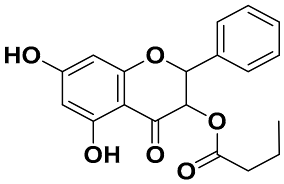

(18)

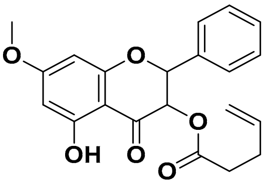

(19)

Figure S1. Compounds **1–19** found in Colombian propolis extracts.
